# Strain-specific anti-biofilm and antibiotic-potentiating activity of 3′,4′-difluoroquercetin

**DOI:** 10.1038/s41598-020-71025-7

**Published:** 2020-08-25

**Authors:** Wonyoung Kho, Mi Kyoung Kim, Minji Jung, Yong Pil Chong, Yang Soo Kim, Ki-Ho Park, Youhoon Chong

**Affiliations:** 1 Department of Integrative Bioscience and Biotechnology, Bio/Molecular Informatics Center, Konkuk University, 120 Neungdong-ro, Gwangjin-gu, Seoul, 05029 Republic of Korea; 2 Department of Infectious Disease, Kyung Hee University School of Medicine, Seoul, Republic of Korea; 3 Department of Infectious Diseases, Asan Medical Center, University of Ulsan College of Medicine, Seoul, Republic of Korea

**Keywords:** Antibiotics, Bacterial infection

## Abstract

Antibacterial properties of 3′,4′-difluoroquercetin (di-F-Q), a fluorine-substituted stable quercetin derivative, were investigated. Even though di-F-Q itself did not show interesting antibacterial activity, treatment of the *Staphylococcus aureus* strains with di-F-Q resulted in a dose-dependent reduction in biofilm formation with IC_50_ values of 1.8 ~ 5.3 mg/L. Also, the antibacterial activity of ceftazidime (CAZ) against carbapenem-resistant *Pseudomonas aeruginosa* (CRPA) showed eightfold decrease upon combination with di-F-Q. Assessment of the antimicrobial activity of CAZ in combination with di-F-Q against 50 clinical isolates of *P. aeruginosa* confirmed 15.7% increase in the percentages of susceptible *P. aeruginosa* isolates upon addition of di-F-Q to CAZ. Further mechanistic studies revealed that di-F-Q affected the antibiotics efflux system in CRPA but not the β-lactamase activity. Thus, di-F-Q was almost equally effective as carbonyl cyanide *m*-chlorophenyl hydrazine in inhibiting antibiotic efflux by *P. aeruginosa*. In vivo evaluation of the therapeutic efficacy of CAZ-(di-F-Q) combination against *P. aeruginosa* showed 20% of the mice treated with CAZ-(di-F-Q) survived after 7 days in IMP carbapenemase-producing multidrug-resistant *P. aeruginosa* infection group while no mice treated with CAZ alone survived after 2 days. Taken together, di-F-Q demonstrated unique strain-specific antimicrobial properties including anti-biofilm and antibiotic-potentiating activity against *S. aureus* and *P. aeruginosa*, respectively.

## Introduction

**Figure 1:**
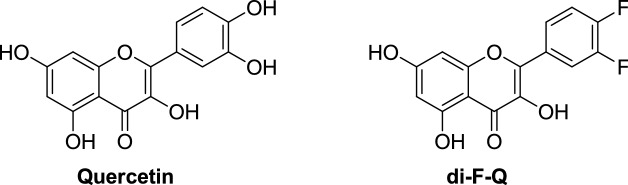
Structures of quercetin and di-F-Q^3^.

Quercetin (Fig. 1) is one of the most famous flavonoids, and it is characterized by broad spectrum biological activity including antioxidant, anti-inflammatory, antiviral, and anticancer effects^1^. Quercetin has also been reported to have a potential as an antibacterial agent^1^, and its anti-biofilm activity against clinical isolates of gram-positive bacteria, albeit low^2^, is noteworthy. However, several unfavorable physicochemical properties of quercetin such as low chemical stability limits its pharmaceutical use. Previously, in order to tackle this problem, we prepared 3′,4′-difluoroquercetin (di-F-Q, Fig. 1) through bioisosteric replacement of the catecholic hydroxyl groups of quercetin with fluorine atoms, which showed significantly improved chemical stability as well as anticancer activity compared with quercetin^3^. In this context, antibacterial properties of di-F-Q appears worth investigating and, in this study, we attempted to evaluate antibacterial, anti-biofilm, and antibiotic-potentiating activity of di-F-Q.

## Materials and methods

### Materials

Dimethyl sulfoxide (DMSO), crystal violet and phosphate buffered saline (PBS) were purchased from Merck (St. Louis, MO, USA). Mueller–Hinton broth (MHB) and tryptic soy broth (TSB) were obtained from BD Biosciences (San Jose, CA, USA) and Fisher Scientific (Pittsburgh, PA, USA), respectively.

### Microorganisms

The bacterial strains used in this study are listed in Table 1.

**Table 1:** Bacterial strains.

Class	Microorganisms	Antimicrobial susceptibility	Source	Strain designation (equivalent designation)	Strain characteristics	References
Gram-positive	*Staphylococcus aureus*	MSSA	ATCC	ATCC 29213		^4^
		MRSA	AMC	AMCSA 5016 (ST5)	Sequence type 5, SCC*mec* II strain, *agr* functional	^5^
		MRSA	AMC	AMCSA 5013 (ST72)	Sequence type 72, SCC*mec* IV, *agr* functional	^5^
		MRSA	AMC	AMCSA 3416 (ST239)	Sequence type ST239, SCC*mec* III, *agr* dysfunctional	^5^
		hVISA	ATCC	ATCC 700698 (Mu3)		^6^
		VISA	ATCC	ATCC 700699 (Mu50)		^6^
	*Enterococcus* species	VSE	ATCC	ATCC29212		^7^
		VRE	KNRRC	CCARM5024		^8^
Gram-negative	*Pseudomonas aeruginosa*	CSPA	ATCC	ATCC27853		^9^
		CRPA	KNRRC	CCARM2321		^10^
	*Acinetobacter baumannii*	CSAB	ATCC	ATCC19606		^11^
		CRAB	AMC	AMC-AB 643		^10^
	*Klebsiella pneumoniae*	wild type	ATCC	ATCC13883		^12^
		CRE	AMC	AMC-KP24272	*K. pneumoniae* carbapenemase (KPC)-producing strain	^10^
	*Escherichia coli*	wild type	ATCC	ATCC25922		^13^
		CRE	AMC	AMC-EC22365	New Delhi Metallo-β-lactamase (NDM-1) producing strain	^10^

*MSSA* methicillin sensitive *Staphylococcus aureus*, *MRSA* methicillin resistant *Staphylococcus aureus*, *hVISA* heteroresistant vancomycin-intermediate *Staphylococcus aureus*, *VISA* vancomycin-intermediate *Staphylococcus aureus*, *VSE* vancomycin-sensitive *Enterococcus faecium*, *VRE* vancomycin-resistant *Enterococcus faecalis*, *CSPA* carbapenem-susceptible *Pseudomonas aeruginosa*, *CRPA* carbapenem-resistant *Pseudomonas aeruginosa*, *CSAB* carbapenem-susceptible *Acinetobacter baumannii*, *CRAB* carbapenem-resistant *Acinetobacter baumannii*, *CRE* carbapenem-resistant *Enterobacteriaceae*, *ATCC* American Type Culture Collection (Manassas, VA, USA), *AMC* Asan Medical Center (Seoul, Korea), *KNRRC* Korea National Research Resource Center (Seoul, Korea).

### Assessment of antibacterial activity of di-F-Q

Minimum inhibitory concentration (MIC) of di-F-Q was determined for all isolates in three replicates using broth microdilution with cation-adjusted MHB according to the Clinical and Laboratory Standards Institute (CLSI) guidelines^14^. DMSO stock solution of di-F-Q was serially diluted to the desired concentrations in MHB. Then, 10 μl of the bacterial suspension [5 × 10^5^ colony forming units (CFU)/ml] was combined with 200 μl of di-F-Q in 96-well microtiter plates (1% DMSO, final). After incubation of the plate at 37 °C for 24 h, bacterial growth was visibly evaluated by monitoring the turbidity of the resulting suspension, and the MICs were determined as the lowest di-F-Q concentration that inhibited the visible growth of the bacteria.

### Anti-biofilm activity

The inhibitory effect of di-F-Q on biofilm formation of various bacterial strains was determined by 96-well plate-based crystal violet assay as described previously^15^. Bacterial strains were cultured with 0.5 × TSB and diluted to 5 × 10^5^ CFU/ml in fresh 0.5 × TSB with 1% glucose. In a 96-well plate, 10 μl of di-F-Q was mixed with 190-μl of the bacterial suspension to final concentrations of 0, 1, 5, 10, 25, 50, and 100 mg/l. The plate was incubated for 24 h and gentle aspiration of media was followed by fixing the biofilm by addition of 100% ethanol. Ethanol was then removed, and the plate was air-dried. The wells were treated with 0.1% crystal violet for 10 min. The plates were washed with deionized water (dH_2_O), and the crystal violet-stained biofilms were solubilized with 33% acetic acid for 1 h. Absorbance of the resulting solution at 600 nm was monitored.

### Antibiotic resistance potential

Antibacterial activity (MICs) of various antibiotics including ampicillin (AMP), ceftazidime (CAZ), cefepime (FEP), meropenem (MER), and vancomycin (VAN) were evaluated in the absence and presence of di-F-Q by using the checkerboard synergy test^16,17^. A total of 100 μl of cation-adjusted MHB was distributed in each well of the 96-well microtiter plate. Di-F-Q was then serially diluted along the ordinate of the plate (0.25 ~ 128 μg/ml), while the second antibiotic was diluted along the abscissa (0.06 ~ 512 μg/ml). From each bacterial isolate, an inoculum standardized with 0.5 McFarland turbidity standard was prepared in cation-adjusted MHB. Each microtiter well was inoculated with 200 μl of a bacterial inoculum (5 × 10^5^ CFU/ml), and the plates were incubated at 35 °C for 20 h under aerobic conditions. The MIC values of each antibiotics in combination with di-F-Q was determined as described above. At least three independent experiments were performed for each strain.

### Inhibition of β-lactamase

Inhibitory activity of di-F-Q against the β-lactamase activity was explored by using a β-lactamase Inhibition Screening Assay Kit (K804-100) (Biovision, San Francisco, USA) which includes nitrocefin, a β-lactamase substrate generating a colorimetric species, as a marker for β-lactamase activity. Assay was performed in accordance with the manufacturer’s instructions, and changes in absorbance A_490_ for nitrocefin was monitored. Clavulanic acid was used as a positive control.

### Inhibition of efflux pumps

The efflux activity of CRPA was assessed by determining the accumulation of ethidium bromide (EtBr) as described previously^18^. Overnight culture of CRPA strain was inoculated in MHB. After incubation at 37 °C for 4 h, the bacterial cells were centrifuged (3,000 rpm, 15 min) and the pellet was resuspended in PBS. After adjusting the OD_600_ to 0.1, the bacterial suspension (186 µl) was added to each well of the flat bottomed, black 96-well plate. EtBr (10 µl) was also added to each well to a final concentration of 2.5 µM. Finally, 4 µl of di-F-Q or a positive control (carbonyl cyanide *m*-chlorophenyl hydrazine, CCCP) was added to the plate and the bacteria were incubated at 37 °C. The fluorescence emission from EtBr was taken (λ_ex_ 535 nm/λ_em_ 600 nm) for 30 min with a 1-min interval using Cytation 5 imaging multi-mode reader (BioTek Instruments, Inc., Winooski, VT, USA).

### In vivo antibacterial activity of CAZ in combination with di-F-Q

Animal experiments were carried out in accordance with established practices as described in the National Institutes of Health Guide for Care and Use of Laboratory Animals and approved by the Institutional Animal Care and Use Committee of the Asan Institute for Life Sciences, Seoul, Korea. Six-week-old female outbred immunocompetent CD-1 mice were randomly distributed into 2 groups (10 mice per group), which were infected with 1.0 × 10^7^ CFU/mouse of *P. aeruginosa* ATCC 27853 (CSPA) or IMP carbapenemase-producing MRPA admixed with 5% porcine mucin. The mice of each group were further allocated in two subgroups (n = 5 for each subgroup) and, after 2 h, the mice in each subgroup were treated with CAZ (10 mg/kg/day) or CAZ-(di-F-Q) (10 mg/kg/day–40 mg/kg/day) for 7 days.

### Statistical analysis

Data are representative of at least three independent experiments and expressed as means ± SD. Tests used for nonparametric data included one-way analysis of variance (ANOVA) with Tukey’s post hoc test (GraphPad, Prism 5).

## Results and discussion

### Antimicrobial activity of di-F-Q

The antibacterial activity of di-F-Q against various gram-positive and gram-negative bacterial strains was investigated (Table 1). Gram-positive bacteria included six *S. aureus* strains [MSSA, MRSA with different sequence types (ST 5, ST72, and ST239), hVISA, and VISA] and two *Enterococcus* strains (VSE and VRE) (Table 1). Eight gram-negative bacterial strains [*P. aeruginosa* (CSPA and CRPA), *A. baumannii* (CSAB and CRAB), *K. pneumoniae* (wild type and CRE KPC type), and *E. coli* (wild type and CRE NDM-1)] were also tested for their susceptibility to di-F-Q by using the broth microdilution checkerboard (CB) method (Table 1). The gram-positive strains were weakly sensitive to di-F-Q (MIC, 8–32 mg/L), but gram-negative strains were highly resistant (MIC, > 128 mg/L) (Table 2). This difference may be explained by the structure and composition of bacterial cell wall. Gram-positive bacteria consist of a relatively simple envelope, and its cytoplasmic membrane is surrounded by peptidoglycan layer. Previously, sub-MICs of quercetin and its derivatives were shown to disrupt or alter cytoplasmic membrane to exhibit antibacterial activity against *S. aureus*^19,20^. Based on the structural similarity, di-F-Q is presumed to have the similar mode of action as quercetin against gram-positive bacteria. On the other hand, gram-negative bacterial cell envelope is more complex and relatively impermeable structure due to their distinctive outer membrane containing lipopolysaccharide^21^. As a result, gram-negative bacteria are protected from many antibiotics, especially large and hydrophobic ones, to which gram-positive bacteria are susceptible. The lack of antibacterial activity of di-F-Q against the gram-negative bacteria might be attributed to its hydrophobic nature (cLogP = 3.78) and resulting inability to cross the bacterial cell wall. In this context, it is worth to note that Wang et al. reported that higher concentration of quercetin (50 × MIC) was required to damage the membrane of *E. coli* compared with *S. aureus* (10 × MIC)^20^.

**Table 2:** MICs (mg/l) of di-F-Q against various gram-positive and gram-negative bacteria.

Gram-positive Bacteria		MIC (mg/l)	Gram-negative Bacteria		MIC (mg/l)
Species	Sensitivity		Species	Sensitivity	
*S. aureus*	MSSA	16	*P. aeruginosa*	CSPA	> 128
	MRSA (ST5)	16		CRPA	> 128
	MRSA (ST72)	16	*A. baumannii*	CSAB	> 128
	MRSA (ST239)	8		CRAB	> 128
	hVISA	16	*K. pneumoniae*	WT	> 128
	VISA	8		CRE KPC	> 128
*Enterococcus* species	VSE	32	*E. coli*	WT	> 128
	VRE	16		CRE NDM-1	> 128

### Anti-biofilm activity of di-F-Q

**Figure 2:**
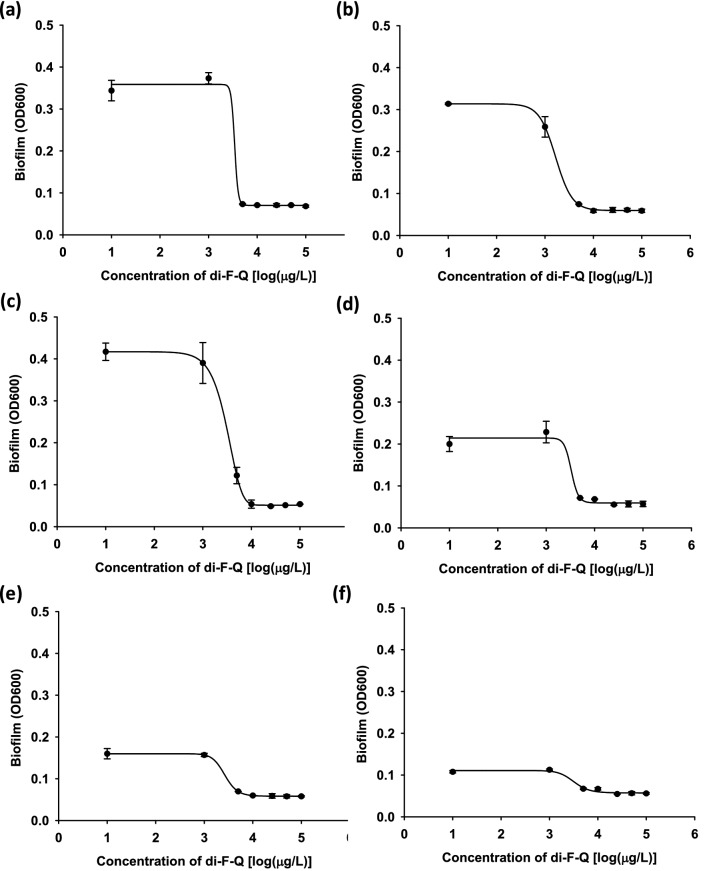
Concentration-dependent inhibitory effect of di-F-Q on the formation of biofilms of (**a**) MSSA, (**b**) MRSA (ST-5), (**c**) MRSA (ST-72), (**d**) MRSA (ST-239), (**e**) hVISA and (**f**) VISA; n = 3, ± SD.

The anti-biofilm activity of di-F-Q was then investigated because, by using this protective mechanism^22,23^, bacteria are known to become significantly more resistant to antibiotics^24^. The bacterial strains tested above were monitored for their biofilm-formation activity in the presence of increasing concentrations of di-F-Q, and a noticeable change was observed only in the *S. aureus* strains which are well characterized for pronounced ability to form biofilms^25^ (Fig. 2). Treatment of the six *S. aureus* strains with di-F-Q resulted in a noticeable dose-dependent reduction in biofilm formation with IC_50_ values of 1.8 ~ 5.3 mg/l (Table 3). As mentioned above, quercetin and its derivatives more easily disrupt the membrane of gram-positive bacteria compared with that of gram-negative bacteria. In addition, previous studies showed that quercetin and its derivatives inhibit hemolysis and biofilm formation in *S. aureus* by reducing alpha-toxin secretion^26–29^, which might explain the *S. aureus*-specific anti-biofilm effect of di-F-Q. On the other hand, quercetin is also known to inhibit the *aqr* quorum-sensing system which modules biofilm formation in *S. aureus*^28^. However, in current investigation, anti-biofilm effect of di-F-Q was observed in both *agr*-functional MRSA strains (AMC-SA 5013, AMC-SA 5016) and *agr*-dysfunction MRSA strain (AMC-SA 3416). In addition, although quercetin exerted strong anti-biofilm effects on *P. aeruginosa* stain PAO1 by repressing expression levels of quorum-sensing associated genes^30^, di-F-Q did not exert anti-biofilm effect on our *P. aeruginosa* strains (data not shown). Taken together, it seems that inhibition of quorum-sensing system may not be essential for anti-biofilm effect of di-F-Q.

**Table 3:** IC_50_’s (mg/l) of di-F-Q for inhibition of biofilm formation by *S. aureus.*

	MSSA	MRSA (ST-5)	MRSA (ST-72)	MRSA (ST-236)	hVISA	VISA
IC_50_ (mg/l)	3.3	1.8	4.5	4.3	3.9	5.3

### Antibiotics-potentiating activity of di-F-Q

The potent anti-biofilm activity of di-F-Q prompted further investigation on its antibiotic properties particularly in combination with other antibiotics. Thus, antibiotic-potentiating activity of di-F-Q was evaluated and, in the presence of sub-MICs (MIC/4) of di-F-Q, antibacterial activity of ampicillin (AMP, aminopenicillin), ceftazidime (CAZ, 3rd generation cephalosporin), cefepime (FEP, 4th generation cephalosporin), meropenem (MEM, carbapenem) and vancomycin (VAN, glycopeptide) were determined against various antibiotic-resistant gram-positive and gram-negative bacteria (Table 4). In general, the antibacterial activity of the antibiotics was not significantly affected by combination with di-F-Q. To our surprise, however, MICs of CAZ against the gram-negative carbapenem-resistant strains such as CRPA and CRAB showed significant decreases (eight- and four-fold, respectively) upon combination with di-F-Q (MIC/4, Table 4).

**Table 4:** MICs (mg/l) of various antibiotics against antibiotic-resistant gram-positive and gram-negative bacteria in the absence and presence of di-F-Q.

Antibiotics	Conc. of di-F-Q	MICs (mg/l) against various bacterial strains^a^						
		Gram-positive			Gram-negative			
		MRSA (ST72)	hVISA	VRE	CRPA	CRAB	CRE KPC	CRE NDM-1
AMP	0	16	64	256	–^b^	–^b^	–^b^	–^b^
	MIC/4	16	32	128	–^b^	–^b^	–^b^	–^b^
CAZ	0	256	> 256	> 256	16	256	> 256	> 256
	MIC/4	256	> 256	> 256	2^c^	64^c^	> 256	> 256
FEP	0	64	> 256	> 256	64	64	256	8
	MIC/4	64	> 256	> 256	32^c^	64^c^	256	8
MER	0	–^b^	–^b^	–^b^	16	64	256	4
	MIC/4	–^b^	–^b^	–^b^	16	64	256	4
VAN	0	0.5	1	> 256	–^b^	–^b^	–^b^	–^b^
	MIC/4	0.5	1	> 256	–^b^	–^b^	–^b^	–^b^

^a^Shaded cells indicate MICs of the antibiotics decreased more than fourfold upon combination with di-F-Q.

^b^Not determined.

^c^Conc. of di-F-Q = 32 mg/L.

**Figure 3:**
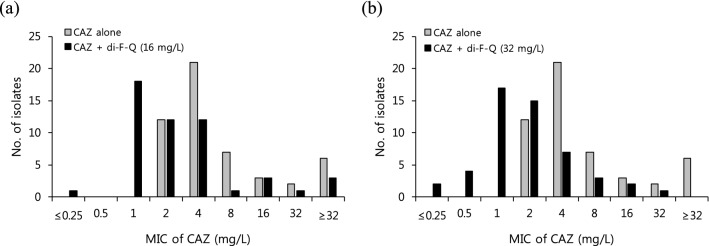
MIC distributions of CAZ for *P. aeruginosa* (n = 50) in the absence and presence of (**a**) 16 mg/l and (**b**) 32 mg/l of di-F-Q. At least three independent experiments were performed for each strain.

The remarkable effect of di-F-Q on the selective restoration of the antibacterial activity of CAZ against carbapenem-resistant gram-negative strains such as CRPA and CRAB is of critical interest because carbapenems are used as last resorts for the treatment of infections caused by multidrug-resistant gram-negative bacteria^31^. Thus, the antimicrobial activity of CAZ alone and in combination with di-F-Q was further assessed against 50 clinical isolates of *P. aeruginosa* using broth microdilution method. The MIC distributions of CAZ against *P. aeruginosa* isolates were obtained in the absence and presence of di-F-Q, which showed significant shifts to lower values upon combination with di-F-Q (Fig. 3). The ceftazidime-potentiating activity of di-F-Q and thereby sensitization of the *P. aeruginosa* isolates to CAZ was found to be concentration-dependent, and the shifts in MICs were more pronounced with increasing concentrations of di-F-Q (Fig. 3).

Based on the Clinical and Laboratory Standards Institute (CLSI) susceptibility interpretive criteria (breakpoints) for CAZ against *P. aeruginosa*^14^, the CAZ-(di-F-Q) susceptibility testing results against 50 *P. aeruginosa* isolates were categorized into percentages of susceptible (%S, MIC ≤ 8), intermediate (%I, MIC = 16) and resistant (%R, MIC ≥ 32) in Table 4. Also included in Table 4 are the MIC_50_ and the MIC_90_ values, the lowest concentration of CAZ at which 50% and 90% of the isolates were inhibited, respectively. CAZ-(di-F-Q) activity (MIC_50/90_, 2/8 mg/l; 94.1% susceptible at 8 mg/l) against all 50 *P. aeruginosa* isolates (with the di-F-Q concentration fixed at 32 mg/l) was noticeably enhanced in comparison to the CAZ single treatment (MIC_50/90_, 4/ > 32 mg/l; 78.4% susceptible at 8 mg/l), which amounts to 15.7% increase in the percentages of susceptible *P. aeruginosa* isolates upon addition of di-F-Q to CAZ (Table 5).

**Table 5:** Antimicrobial activity of ceftazidime (CAZ) alone and in combination with di-F-Q against 50 clinical isolates of *P. aeruginosa.*

Antimicrobial Agent	MIC_50_ (mg/l)^a^	MIC_90_ (mg/l)^b^	CLSI^c^ breakpoint^d^		
			%S	%I	%R
CAZ alone (n = 50)	4	> 32	78.4	5.9	15.7
CAZ + di-F-Q (32 mg/L) (n = 50)	2	8	94.1	3.9	2.0

^a^MIC required to inhibit 50% of isolates.

^b^MIC required to inhibit 90% of isolates.

^c^Clinical Laboratory Standards Institute.

^d^S (Sensitive): MIC ≤ 8, I (Intermediate): MIC = 16, R (Resistant): MIC ≥ 32 (mg/l).

**Figure 4:**
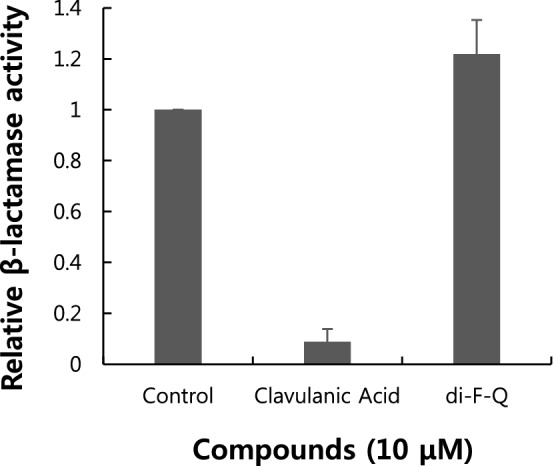
Relative β-lactamase activity after treatment with 10 μM of clavulanic acid (a positive control) and di-F-Q; n = 3, ± SD.

The significantly increased sensitization of *P. aeruginosa* isolates to CAZ by di-F-Q is reminiscent of CAZ-avibactam (AVI) combination (MIC_50/90_, 2/8 mg/l; 96.3% susceptible at 8 mg/l) which showed 7.4 ~ 16.3% increases in the percentages of the susceptible *P. aeruginosa* isolates compared with those of CAZ single treatment^32,33^. As the CAZ-potentiation activity of avibactam is attributed to its β-lactamase inhibitory activity^33^, we evaluated the effect of di-F-Q on the enzymatic activity of β-lactamase. However, the β-lactamase activity was not affected by treatment with di-F-Q (Fig. 4).

**Figure 5:**
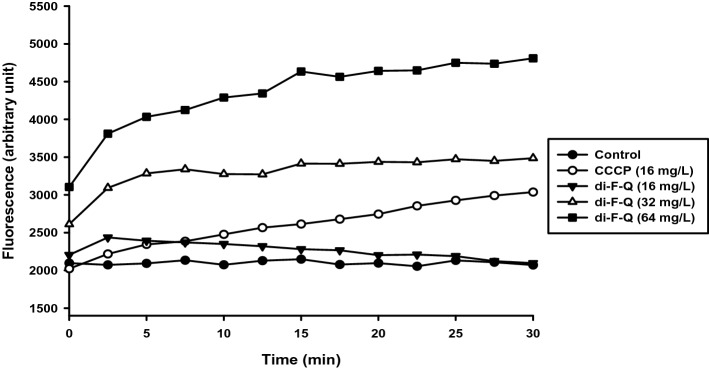
Fluorescence emission from EtBr in CRPA after treatment with CCCP (16 mg/l) and di-F-Q (16, 32, and 64 mg/l).

On the other hand, overexpression of efflux pumps is generally considered as the major mechanism for intrinsic and acquired antibiotic resistance in *P. aeruginosa*^34^; a diverse array of antibiotics including β-lactams, quinolones, tetracyclines, macrolides, linomycins chloramphenicols and novobiocins are known to suffer from efflux pump-mediated resistance by *P. aeruginosa*^35^. Thus, efflux pump inhibitory activity of di-F-Q was investigated by using ethidium bromide (EtBr) as the fluorescent substrate of efflux pumps in *P. aeruginosa* (Fig. 5)^36^. In CRPA, di-F-Q increased EtBr fluorescence in a dose-dependent manner indicating inhibition of efflux pumps and thereby intracellular retention of EtBr; significant efflux pump inhibitory activity of di-F-Q was observed at concentrations 32 mg/l or above, which is in line with the eightfold reduction of the MIC of CAZ against CRPA by the same amount of di-F-Q (Table 3). Carbonyl cyanide m-chlorophenyl hydrazine (CCCP) is known to inhibit the efflux pump in *P. aeruginosa*^36^, and we observed that treatment of CRPA with CCCP (16 mg/l) increased fluorescence from EtBr (Fig. 5). These results collectively suggest that di-F-Q (32 mg/l) and CCCP (16 mg/l) are almost equally effective in inhibiting antibiotic efflux by *P. aeruginosa*. The MexAB-OprM multidrug efflux pump system is known to pump out mostly lipophilic and amphiphilic drugs, which include EtBr.

**Figure 6:**
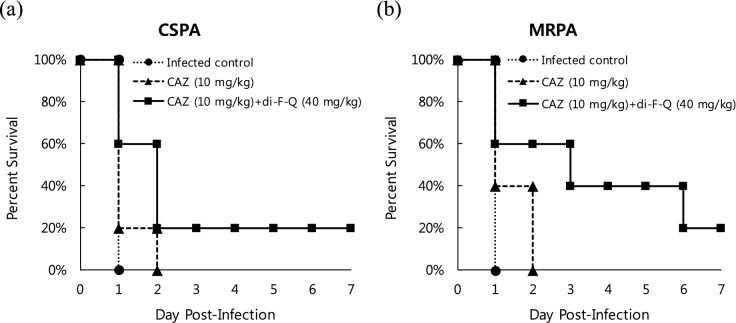
Kaplan–Meier survival curves for mice treated with CAZ (10 mg/kg) with and without di-F-Q (40 mg/kg) after infection with (**a**) CSPA and (**b**) MRPA (n = 5 for each treatment group).

Therapeutic efficacy of CAZ-(di-F-Q) combination against *P. aeruginosa* was then evaluated in mice infected with CSPA or multidrug resistant *P. aeruginosa* (MRPA) (Fig. 6). Thus, six-week-old female outbred immunocompetent CD-1 mice were infected with 1.0 × 10^7^ CFU/mouse of *P. aeruginosa* ATCC 27853 (CSPA) (Fig. 6a) or MRPA (Fig. 6b) admixed with 5% porcine mucin and, after 2 h, treated with CAZ (10 mg/kg/day) or CAZ-(di-F-Q) (10–40 mg/kg/day) for 7 days (n = 5 for each treatment group). The CSPA- or MRPA-infected mice without antibiotic treatment all died after 1 day (infected control, Fig. 6). Also, in both CSPA and MRPA infection groups, no mice treated with CAZ alone survived after 2 days (CAZ, Fig. 6). However, 20% of the mice treated with CAZ-(di-F-Q) survived after 7 days in CSPA as well as MRPA infection group [CAZ + (di-F-Q), Fig. 6]. Interestingly, between 3 to 5 days after infection, CAZ-(di-F-Q) was more effective in protecting MRPA-infected mice (60 ~ 40% survival rate, Fig. 6b) than the CSPA-infected ones (20% survival rate, Fig. 6a).

In summary, we evaluated antibacterial properties of di-F-Q, a fluorinated quercetin analogue. Di-F-Q was not effective in killing bacteria but showed potent anti-biofilm activity against *S. aureus* with IC_50_ values of 1.8 ~ 5.3 mg/l. Also, di-F-Q potentiated the antibacterial activity of the 3rd generation cephalosporin CAZ against the gram-negative carbapenem-resistant strains such as CRPA and CRAB (eight- and four-fold decreases in MICs, respectively). Assessment of the antimicrobial activity of CAZ in combination with di-F-Q against 50 clinical isolates of *P. aeruginosa* confirmed a remarkable enhancement in antibacterial activity (MIC_50/90_, 2/8 mg/l; 94.1% susceptible at 8 mg/l) in comparison to the CAZ single treatment (MIC_50/90_, 4/ > 32 mg/l; 78.4% susceptible at 8 mg/l), which amounts to 15.7% increase in the percentages of susceptible *P. aeruginosa* isolates upon addition of di-F-Q to CAZ. Further mechanistic studies revealed that di-F-Q affected the antibiotics efflux system in CRPA but not the β-lactamase activity. Thus, di-F-Q was almost equally effective as CCCP in inhibiting antibiotic efflux by *P. aeruginosa*. In vivo evaluation of the therapeutic efficacy of CAZ-(di-F-Q) combination against *P. aeruginosa* showed 20% of the mice treated with CAZ-(di-F-Q) survived after 7 days in MRPA infection group while no mice treated with CAZ alone survived after 2 days. Taken together, di-F-Q demonstrated the peculiar strain-specific anti-biofilm and antibiotic-potentiating activity against *S. aureus* and *P. aeruginosa*, respectively.
